# Analysis of a *c*_0_*t-1 *library enables the targeted identification of minisatellite and satellite families in *Beta vulgaris*

**DOI:** 10.1186/1471-2229-10-8

**Published:** 2010-01-11

**Authors:** Falk Zakrzewski, Torsten Wenke, Daniela Holtgräwe, Bernd Weisshaar, Thomas Schmidt

**Affiliations:** 1 Institute of Botany, Dresden University of Technology, D-01062 Dresden, Germany; 2 Institute of Genome Research, University of Bielefeld, D-33594 Bielefeld, Germany

## Abstract

**Background:**

Repetitive DNA is a major fraction of eukaryotic genomes and occurs particularly often in plants. Currently, the sequencing of the sugar beet (*Beta vulgaris*) genome is under way and knowledge of repetitive DNA sequences is critical for the genome annotation. We generated a *c*_0_*t-1 *library, representing highly to moderately repetitive sequences, for the characterization of the major *B. vulgaris *repeat families. While highly abundant satellites are well-described, minisatellites are only poorly investigated in plants. Therefore, we focused on the identification and characterization of these tandemly repeated sequences.

**Results:**

Analysis of 1763 *c*_0_*t-1 *DNA fragments, providing 442 kb sequence data, shows that the satellites pBV and pEV are the most abundant repeat families in the *B. vulgaris *genome while other previously described repeats show lower copy numbers. We isolated 517 novel repetitive sequences and used this fraction for the identification of minisatellite and novel satellite families. Bioinformatic analysis and Southern hybridization revealed that minisatellites are moderately to highly amplified in *B. vulgaris*. FISH showed a dispersed localization along most chromosomes clustering in arrays of variable size and number with exclusion and depletion in distinct regions.

**Conclusion:**

The *c*_0_*t-1 *library represents major repeat families of the *B. vulgaris *genome, and analysis of the *c*_0_*t-1 *DNA was proven to be an efficient method for identification of minisatellites. We established, so far, the broadest analysis of minisatellites in plants and observed their chromosomal localization providing a background for the annotation of the sugar beet genome and for the understanding of the evolution of minisatellites in plant genomes.

## Background

Repetitive DNA makes up a large proportion of eukaryotic genomes [1]. Major findings in the last few years show that repetitive DNA is involved in the regulation of heterochromatin formation, influences gene expression or contributes to epigenetic regulatory processes [2-7]. Therefore, understanding the role of repetitive DNA and the characterization of their structure, organization and evolution is essential. A rapid procedure to identify repetitive DNA is based on *c*_0_*t *DNA isolation [8], which is an efficient method for the detection of major repetitive DNA fractions as well as for the identification of novel repetitive sequences in genomes [9]. The *c*_0_*t *DNA isolation is based on the renaturation of denaturated genomic DNA within a defined period of time and concentration. The rate at which the fragmented DNA sequences reassociate is proportional to the copy number in the genome [8] and therefore, *c*_0_*t *DNA isolated after short reassociation time (e.g. *c*_0_*t-1*) represents the repetitive fraction of a genome. Recently, analyses of *c*_0_*t *DNA were performed in plants e.g. for *Zea mays*, *Musa acuminata*, *Sorghum bicolor *and *Leymus triticoides *[8,10-12].

Satellite DNA consisting of tandemly organized repeating units (monomers) of relatively conserved sequence motifs is a major class of repetitive DNA. Depending on monomer size, tandem repeats are subdivided into satellites, minisatellites and microsatellites and tandem repeats with specific functions such as telomeres and ribosomal genes. The monomer size of minisatellites varies between 6 to 100 bp [13] and those of microsatellites between 2 to 5 bp [14]. Most plant satellites have a monomer length of 160 to 180 bp or 320 to 370 bp [15]. Satellite DNAs are non-coding DNA sequences, which are predominantly located in subterminal, intercalary and centromeric regions of plant chromosomes. The majority of typical plant satellite arrays are several megabases in size [15]. In contrast, arrays of minisatellites vary in length from 0.5 kb to several kilobases [13]. Minisatellites are often G/C-rich and fast evolving [13] and thought to originate from slippage replication or recombination between short direct repeats [16] or slipped-strand mispairing replication at non-contiguous repeats [17]. Minisatellites are poorly investigated in plants. So far, only a few minisatellites were described, for example in *Arabidopsis thaliana*, *O. sativa*, *Triticum aestivum*, *Pisum sativum *and some other plant species [18-26]. Moreover, only two minisatellite families were physically mapped on plant chromosomes using fluorescent *in situ *hybridization (FISH) [19].

The sequencing of the sugar beet (*Beta vulgaris*) genome, which is about 758 Mb in size [27] and has been estimated to contain 63% repetitive sequences [28], is under way and the first draft of genome sequence is currently established [29]. Knowledge about repetitive DNA and their physical localization is essential for the correct annotation of the sugar beet genome. Therefore, we detected and classified the repeated DNA fraction of *B. vulgaris *using sequence data from cloned *c*_0_*t-1 *DNA fragments. We focused on the investigation of novel tandem repeats and characterized nine minisatellite and three satellite families. Their chromosomal localization was determined by multicolor FISH and the organization within the genome of *B. vulgaris *was analyzed by Southern hybridization.

## Results

### *c*_0_*t-1 *analysis reveals the most abundant satellite DNA families of the *B. vulgaris *genome

**Figure 1:**
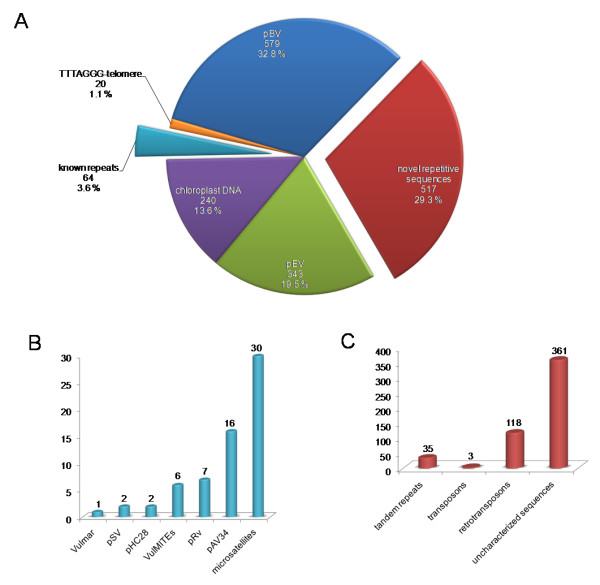
**Classification of isolated *c*_0 _*t-1 *DNA sequences**. **A**: Absolute and relative distribution of 1763 *c*_0_*t*-1 sequences of the *B. vulgaris *genome. **B**: Number of clones (known repeats in A) with similarities to previously described *B. vulgaris *repeats. **C**: Classification of novel repetitive sequences.

In order to analyze the composition of the repetitive fraction of the *B. vulgaris *genome, we prepared *c*_0_*t-1 *DNA from genomic DNA and generated a library consisting of 1763 clones with an average insert size between 100 to 600 bp providing in total 442 kb (0.06% of the genome) sequence data. For the characterization of the *c*_0_*t-1 *DNA sequences we performed homology search against nucleotide sequences and proteins in public databases and classified all clones based on their similarity to described repeats, telomere-like motifs, chloroplast-like sequences as well as novel sequences lacking any homology (Figure 1). More than half of the *c*_0_*t-1 *fraction (60%) belongs to known repeat classes including mostly satellites. In order to determine the individual proportion of each repeat family we applied BLAST analysis using representative query sequences of each repeat. We observed that the relative frequency of repetitive sequence motifs found in the *c*_0_*t-1 *library correlates with its genomic abundance in *B. vulgaris*: The most frequently occurring repeat is pBV (32.8%, 579 clones), [EMBL:Z22849], a highly repetitive satellite family that is amplified in large arrays in centromeric and pericentromeric regions of all 18 chromosomes [30,31]. The next repeat in row has been observed in 19.5% of cases (343 clones) and belongs to the highly abundant satellite family pEV [EMBL:Z22848] that forms large arrays in intercalary heterochromatin of each chromosome arm [32]. The *c*_0_*t-1 *DNA library also enabled the detection of moderately amplified repeats. Telomere-like motifs of the *Arabidopsis*-type were detected in 1.1% (20 clones) while a smaller proportion of sequences belong to the satellite family pAv34 (0.9%, 16 clones), [EMBL:AJ242669] which is organized in tandem arrays at subtelomeric regions [33]. Only 0.1% (2 clones) belong to the satellite families pHC28 [EMBL:Z22816] [34] and pSV [EMBL:Z75011] [35], respectively, which are distributed mostly in intercalary and pericentromeric chromosome regions. Furthermore, microsatellite motifs were found in 1.7% of *c*_0_*t-1 *sequences [36]. Miniature inverted-repeat transposable elements (MITEs) [EMBL:AM231631], derived from the *Vulmar *family of *mariner *transposons [37], were identified in 0.3% (6 clones) of the *c*_0_*t-1 *sequences, while *Vulmar *[EMBL:AJ556159] [38] was detected in a single clone only. The repeat pRv [EMBL:AM944555] was found in a relatively low number of *c*_0_*t-1 *sequences (0.4%, 7 clones) indicating lower abundance than the satellite pBV. pRv is only amplified within pBV monomers and forms a complex structure with pBV [31]. Surprisingly, the homology search enabled the detection of a large amount of *c*_0_*t-1 *sequences (13.6%) that show similarities to chloroplast DNA.

The identification of novel repetitive sequences was an aim of the *c*_0_*t-1 *analysis. Altogether, we identified 29.3% (517 clones) of the *c*_0_*t-1 *sequences lacking homology to previously described *B. vulgaris *repeats. However, to verify the repetitive character of each sequence motif we performed BLAST search against available *B. vulgaris *sequences. 56582 BAC end sequences (BES) [39], (Holtgräwe and Weisshaar, in preparation) covering 5.2% of the genome were used for analysis. 360 *c*_0_*t-1 *sequences showed hits in BES ranging from 11 to 300 while 39 sequences showed more than 300 hits and 118 sequences less than 10 hits. This observation indicates that many of these yet uncharacterized *c*_0_*t-1 *clones contain sequence motifs that are highly to moderately amplified in the genome.

We performed an assembly of the 517 uncharacterized *c*_0_*t-1 *clones to generate contigs, which contain sequences belonging to an individual repeat family. In total, 37 contigs ranging in size from 149 bp to 1694 bp (average size 555 bp) were established. The largest contig in size and clone number (1694 bp, 20 sequences) was used for BLAST search against available sequences. Analysis of the generated alignment revealed a LTR of a retrotransposon. The full-length element designated Cotzilla was classified as an *envelope*-like *Copia *LTR retrotransposon related to sireviruses [40]. The internal region of Cotzilla showed similarity to 40 sequences of 118 *c*_0_*t-1 *clones categorized as retrotransposon-like (Figure 1C) showing that Cotzilla is the most abundant retrotransposon within the *c*_0_*t-1 *library. Analysis of a further contig (1081 bp, 4 clones) resulted in the identification of the LTR of a novel *Gypsy *retrotransposon (unpublished) that shows 13 hits within the *c*_0_*t-1 *library. Three further clones displayed similarities to transposons. The remaining uncharacterized *c*_0_*t-1 *clones (396 sequences) were used for the identification of tandemly arranged repeats.

#### Targeted isolation of minisatellites and satellites using the *c*_0_*t-1 *library

Plant minisatellites do not have typical conserved sequence motifs, therefore the analysis of *c*_0_*t *DNA is a useful method for the targeted isolation of minisatellites. We scanned the 396 clones of the *c*_0_*t-1 *library that show no similarity to known repeats and detected 35 sequences that contain tandemly repeated sequences. Based on their similarity these sequences were grouped into nine minisatellite families and three satellite families. The minisatellites were named according to their order of detection and the satellites according to conserved internal restriction sites (Table 1). A sequence of each tandem repeat family was used as query and blasted against available sequences to identify additional *B. vulgaris *copies. Alignments of all sequences of each tandem repeat family were generated and the average monomer size, the G/C-content and the identity values of at least 20 randomly selected monomers determined (Table 1).

**Table 1:** Minisatellites and satellites identified in the *c*_0 _*t-1 *library of *B. vulgaris*

tandem repeat	size [bp]	*c*_0 _*t-1 *hits	G/C-content [%]	identity [%]	EMBL accession	representative monomere sequence
BvMSat01	10	7	34	40 - 100	ED023089	AACTTATTGG
BvMSat11	15	1	41	36 - 100	DX580797	TAAATAGTCAAGCCC
BvMSat05	21	5	29	38 - 100	ED029002	ACTGAAAAAAAATGAAGACTA
BvMSat07	30	4	32	90 - 100	ED019743	GAAAAAATAAGTTCAGATCAGATCAGATCA
BvMSat08	32	1	48	77 - 100	DX107266	GGGTCGGAATAAATCGGCTTTCGAAATGACTT
BvMSat09	32-39	5	24	46 - 100	FN424406	AGAAGTATACAAGAACATTAATCAAAATATATAAACAAA
BvMSat03	40	3	33	55 - 100	ED024452	GTCTCTAAAGCCATGTATTTAGCGTCACATGAATTTAGTT
BvMSat10	51	3	24	78 - 100	DX980914	GTTTGTTCTTAAAAGGTTGTTCTTGAATTATTATTCAAGTGTTTGGAAAGA
BvMSat04	96	2	41	70 - 100	DX983375	CCTCTAAATGTAAGTGGCTTTAGCAGCACTATAAGTTCTGTGCCTAAAAAA
						GGTGGCATTACGGGCAACCAACAATTAGCGACAGGCATATGGTTG
*Fok*I-satellite	130	1	60	81 - 100	DX979624	GGGACTTAGGAGAGTGACCCAACCAAGGAGGGAGACCTCCTTGGGCTGAGT
						TGGGTGGACGCGGCTCGGATGAGGGGCCAATGAGCCCCACGCTTGTCCGAG
						CCGGTGCCGTCTCTCGCCATGTCAATCT
*Alu*I-satellite	173	1	33	78 - 100	ED022281	ATAATCATACCTCTATGCCTATTCCAAGTTCTAATGGCTAATGCAAGTCCT
						AAAATACTCATTTAAACTTTCTACTACATGGTTGTAAGATTCTAAGCAAGT
						TTAATACACTTAGCCAATTAAAATGAGAAAAACTAAGCCATTTCGAGCCGT
						TTTTTGGGTTTCATGTTCCT
*Hinf*I-satellite	325	2	45	75 - 86	DX982322	TGTGACTTGTAACATTGCGCGGGTGCTTGGCACCATTTGCGTTACCTCAAA
						AAGCCTTTGAACACCCCAATTATTCATTTCTCGCGAAATCCAAAATTGCCT
						CGAAATGAACGTAAAGGCATCCACATATTTGTTCCAAGCCACATGACTCCT
						TTACATTGACCTCCTATGTCCCTAGGAGGCATCCCGTGCCATTTGGAGCTC
						GGGCAACGGGAAAGTCCGAAAGCGTGTATAATCTTCAATTTTAGTTGTTTT
						TGGGGAATTTTTGGACTACTTCTTCAGGCCCGGTCATATTTTTCTTTCGAA
						ACATTCCTAGGAGTGCCGA

The tandem repeats are listed according to their monomer size.

**Figure 2:**
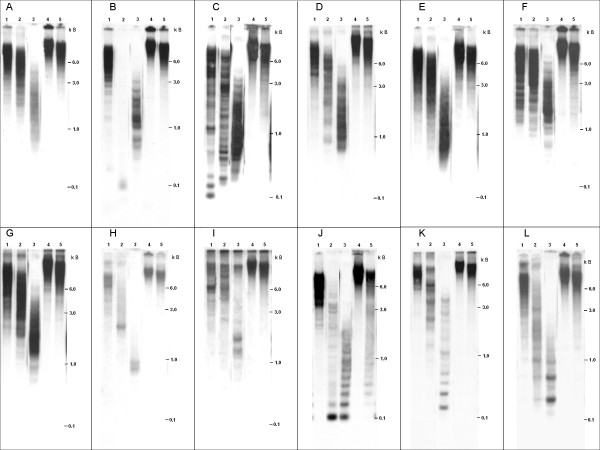
**Southern hybridization of genomic *B. vulgaris *DNA with probes of tandem repeats identified in the *c*_0 _*t-1 *library**. Genomic DNA was restricted with *Nde*I (1), *Bsm*AI (2), *Alu*I (3), *Hpa*II (4) and *Msp*I (5) and hybridized with BvMSat01 (A), BvMSat03 (B), BvMSat04 (C), BvMSat05 (D), BvMSat07 (E), BvMSat08 (F), BvMSat09 (G), BvMSat10 (H), BvMSat11 (I) and the *Fok*I-satellite (J), *Alu*I-satellite (K) and *Hin*fI-satellite (L).

In order to investigate the genomic organization and abundance of the tandem repeats, Southern hybridizations were carried out. A strong hybridization smear of a wide molecular weight range was detected in each case indicating abundance of the minisatellite families in the genome of *B. vulgaris *(Figure 2A - G). Distinct single bands were observed for the minisatellite families BvMSat10 (Figure 2, H) and BvMSat11 (Figure 2, I). Because of the short length, recognition sites for restriction enzymes are rare or absent within minisatellite monomers. Thus, genomic DNA was restricted with 15 different restriction enzymes to identify restriction enzymes generating mono- and multimers in minisatellite arrays detectable by Southern hybridization. Figure 2 illustrates the probing of genomic DNA after restriction with the 5 restriction enzymes generating most ladder-like patterns in minisatellite and satellite arrays. A typical ladder-like pattern is detectable for BvMSat04 (Figure 2C, lane 1) and BvMSat03 (Figure 2B, lane 2). Multiple restriction fragments were observed after hybridization of BvMSat08 (Figure 2F). The tandem organization of the minisatellites lacking restriction sites was confirmed by sequence analysis or PCR (not shown). Typical ladder-like patterns were generated for each satellite family. For example, the tandem organization was verified for the *Fok*I satellite, *Alu*I satellite and *Hin*fI satellite after restriction with *Alu*I (Figure 2, J-L, lane 3,).

To investigate the DNA methylation of the tandem repeats in CCGG motifs, genomic DNA was digested with methylation sensitive isoschizomeres *Hpa*II and *Msp*I. *Hpa*II only cuts CCGG, whereas *Msp*I cuts CCGG and C^met^CGG [41]. We detected very large DNA fragments generated by restriction with *Hpa*II and *Msp*I, which were not resolved by conventional gel electrophoresis indicating reduced restriction of DNA in most minisatellites and adjacent regions (Figure 2, A - I, lane 4 and 5). The DNA methylation of CCGG motifs in *Alu*I and *Hin*fI satellite arrays was observed by the hybridization to very large DNA-fragments (Figure 2, K - L, lane 4 and 5). However, the presence of several small DNA fragments and signals of multimers after restriction with *Msp*I (Figure 2J, lane 5) indicates no CNG methylation of some *Fok*I satellite arrays (Figure 2, J, lane 5).

#### Physical mapping of tandemly repeated *c*_0_*t-1 *clones using FISH

**Figure 3:**
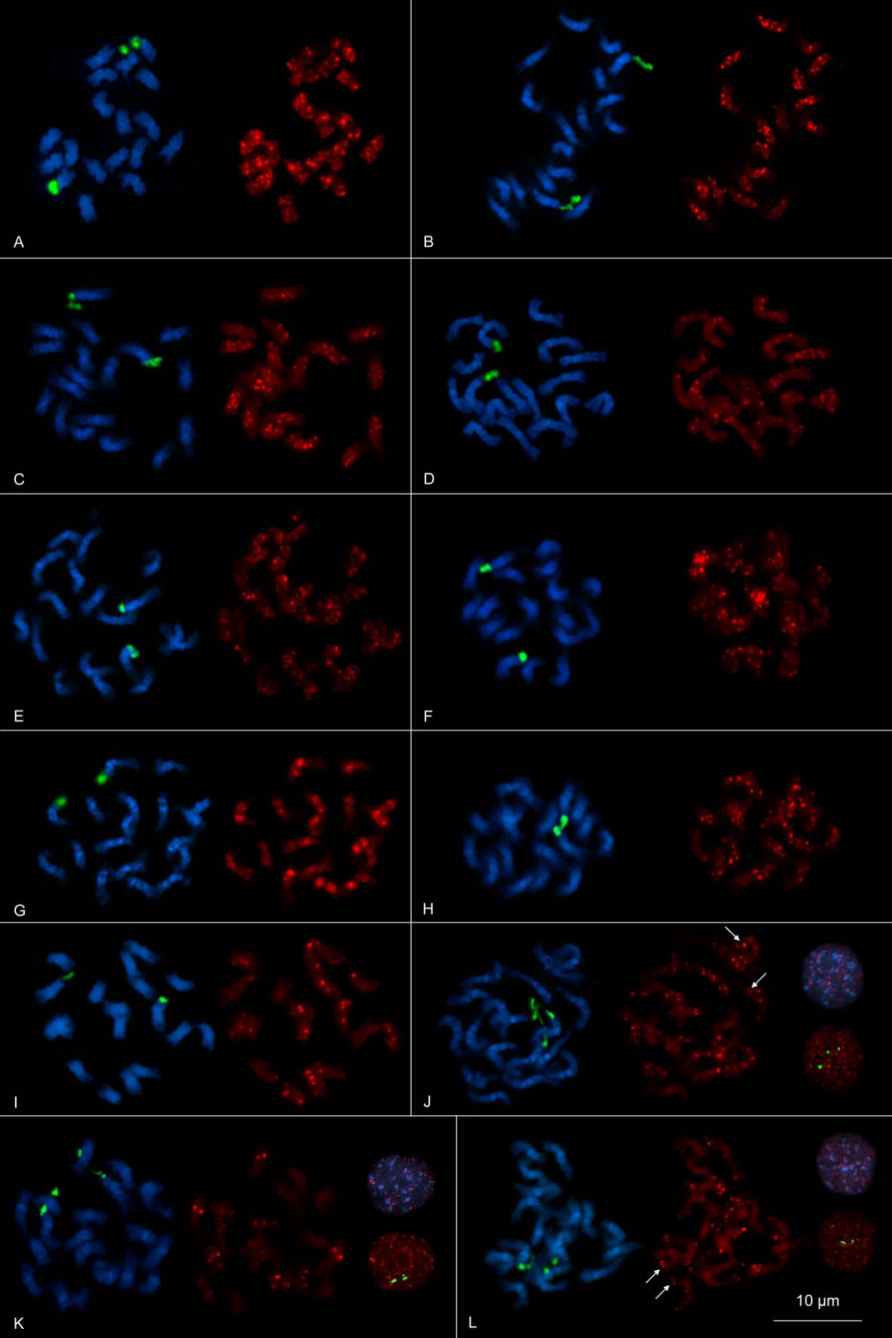
**Physical mapping of tandem repeats on mitotic metaphase chromosomes and interphase nuclei of *B. vulgaris *using FISH**. Blue fluorescence (DAPI stained DNA) shows the morphology of chromosomes. Red signals show chromosomal localization of the tandem repeats and green signals show position of 18S-5.8S-25S rRNA genes on the chromosomes. Hybridization with the minisatellites BvMSat01 (A), BvMSat03 (B), BvMSat04 (C), BvMSat05 (D), BvMSat07 (E), BvMSat08 (F), BvMSat09 (G), BvMSat10 (H), BvMSat11 (I) on mitotic metaphases and probes of the *Fok*I-satellite (J), the *Alu*I-satellite (K) and the *Hin*fI-satellite (L) on mitotic metaphases and interphase nuclei reveals characteristic chromosomal distribution patterns.

The physical distribution of the minisatellite and satellite families on mitotic metaphase chromosomes of *B. vulgaris *was investigated by fluorescent *in situ *hybridization (FISH) (Figure 3). For the visualization of chromosome morphology and structure, metaphase nuclei were stained with DAPI (blue fluorescence in Figure 3). Euchromatin is detectable by less DAPI staining, while stronger intensity indicates heterochromatic regions such as centromeres and pericentromeres. In order to identify chromosome pair 1, metaphase chromosomes were hybridized with 18S-5.8S-25S-rRNA genes (green signals in Figure 3) that show strong signals in terminal regions on one pair of chromosomes. The still decondensed rDNA is displaced or disrupted in some metaphases resulting in additional signals (e.g. Figure 3, K and 3J).

Using minisatellites as probes, similarities in the chromosome distribution patterns were preferentially observed in the intercalary heterochromatin and for some minisatellites in terminal regions as dispersed signals. Only weak signals were detectable in centromeric or pericentromeric regions. Different chromosomes show a variation in signal strength and, hence, in copy numbers or expansion of minisatellite arrays (e.g. Figure 3, A-C, F and 3G). While some chromosomes show stronger banding patterns indicating larger arrays or clustering of multiple arrays, on other chromosomes weak or no signals were revealed (e.g. Figure 3, F and 3G), which shows that minisatellite arrays are often small in size. The detection of signals on both chromatids of many chromosomes verifies the hybridization pattern.

Physical mapping using probes of the minisatellite families BvMSat08 and BvMSat09 shows particular hybridization patterns enabling the discrimination of *B. vulgaris *chromosomes (Figure 3, F and 3G). A peculiar hybridization pattern was observed for BvMSat08, which shows massive amplification of signals in the intercalary heterochromatin (Figure 3, F), which are localized on one chromosome arm of a single chromosome pair indicating very large arrays of multiple BvMSat08 copies or clustering of arrays. Four chromosomes show only reduced signals indicating a lower number of BvMSat08 arrays on these chromosomes. The minisatellite BvMSat09 shows massive accumulation of clusters in the intercalary heterochromatin on twelve chromosomes (Figure 3, G). Six of them are identifiable by blocks on both chromosome arms, whereas the other chromosomes are characterized by blocks on one chromosome arm only.

For the physical mapping of satellites identified in the *c*_0_*t-1 *library we hybridized metaphase chromosomes and also interphase nuclei, which enable the detection of signals at higher resolution (Figure 3, J-L). The *Fok*I-satellite shows a co-localization with DAPI-positive intercalary heterochromatin (Figure 3, J). However, the signals are not uniformly distributed and differ in signal strength. Hybridization was also detected at terminal euchromatic chromosome regions, consistent with the *Fok*I-satellite hybridization pattern in interphase nuclei in low DAPI-stained euchromatic regions (arrows in Figure 3, J).

Strong clustering of *Alu*I-satellite arrays was observed in the intercalary heterochromatin on four chromosomes, while eight chromosomes show a weaker hybridization pattern (Figure 3, K). The remaining six chromosomes show very weak signals indicating that *Alu*I-satellites are also present in low copy numbers. The hybridization pattern in interphase nuclei shows that most *Alu*I-satellite signals are localized within heterochromatic chromosome regions adjacent to euchromatic regions.

Hybridization with probes of the *Hin*fI-satellite shows a different pattern. Signals of the *Hin*fI-satellite are mostly localized in terminal chromosome regions: twelve chromosomes show hybridization on both chromosome arms, while signals only on one chromosome arm are detectable on the remaining six chromosomes (Figure 3, L). Hybridization on interphase nuclei revealed the preferred distribution of *Hin*fI-satellites in euchromatic regions (arrows in Figure 3, L), while only reduced signals are notable in heterochromatic blocks.

#### Minisatellite BvMSat07 consists of a complex microsatellite array

**Figure 4:**
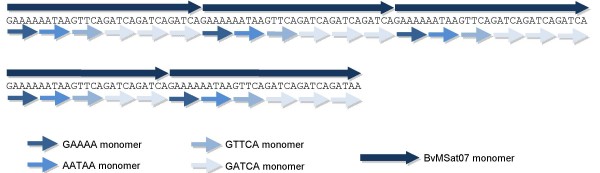
**BvMSat07 is composed of microsatellite complex repeats**. 30 bp monomers of BvMSat07 are typically composed of degenerated and conserved GATCA-motifs (as example an array of the BAC end sequence FN424407 is shown).

Among the *c*_0_*t-1 *sequences, we identified an array of a microsatellite motif with the consensus sequence GATCA. Within several *c*_0_*t-1 *sequences, three short imperfect repeats (GAAAA, AATAA and GTTCA) were interspersed within arrays of GATCA monomers. In order to examine whether this interspersion is conserved, we analyzed *B. vulgaris *sequences possessing GATCA-microsatellite arrays and detected that the minisatellite BvMSat07 is derived from the GATCA-microsatellite. A typical BvMSat07 monomer, which is 30 bp in size, consists of one GAAAA, one AATAA, one GTTCA motif conserved in this order and three adjacent GATCA monomers, respectively (Figure 4). The analysis of 20 randomly selected minisatellite BvMSat07 monomers revealed that most monomers show an identical arrangement of these short subrepeats and that these monomers share a similarity of 90% to 100%.

#### Head to head junction is a typical characteristic of BvMSat05 arrays

The 21 bp minisatellite BvMSat05 varies considerably in nucleotide composition. Sequence identity analysis of 450 monomers originating from *c*_0_*t-1 *and BAC end sequences revealed that monomers show identities between 38% and 100%.

**Figure 5:**
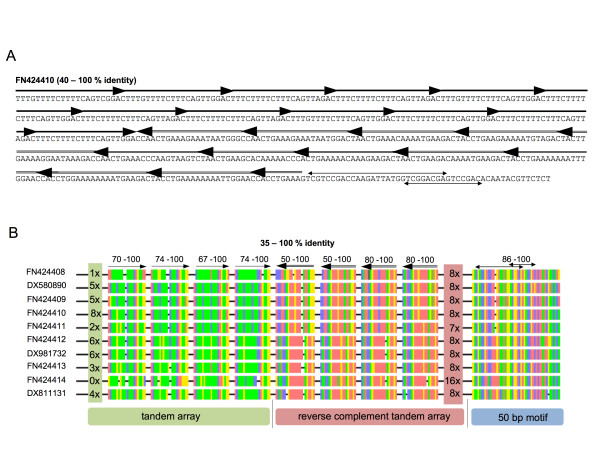
**Illustration of the head to head junction of BvMSat05 arrays**. **A**: The BAC end sequence FN424410 contains a head to head junction of two head to tail BvMSat05 arrays (arrows and double-lined arrows). **B**: An alignment of ten BAC end sequences illustrates the typical head to head junction of two head to tail arrays. For each array four monomers, which are separated by a gap, are shown. The number at the left and right borders of the arrays corresponds to the number of monomers that are not displayed in this illustration. The nucleotides are color-encoded: Red for adenine, blue for cytosine, yellow for guanine and green for thymine. The tandem arrays are flanked one-sided by a highly conserved 50 bp motif, which comprises two palindromic sequences (double arrows). Identity values are displayed in percent.

BvMSat05 shows a particular genomic organization: In addition to the head to tail organization, a head to head junction is detectable within multiple BvMSat05 arrays (Figure 5). Identity values between 35% and 100% of the monomers within the inverted arrangement of the two arrays are similar to the values of head to tail monomers. The tandem arrays of the head to head junction are flanked one-sided by the conserved sequence motif GTCGTCCGACCAAAGATTATGGTCGGACGAGTCCGACACAATACGTTCTCT, which is 50 bp in size and shows identity of 86% to 100% (Figure 5). Interestingly, this sequence comprises two palindromic motifs (TCGTCCGACCAAAGATTATGGTCGGACGA and GTCGGACGAGTCCGAC) (arrows in Figure 5).

## Discussion

The aim of this study was the characterization of the repetitive fraction of the *B. vulgaris *genome. We generated and analyzed 1763 highly and moderately repetitive sequences from a *c*_0_*t-1 *DNA library. Our results revealed that the majority of sequences in the *c*_0_*t-1 *library are copies of the satellite families pBV [30] and pEV [32] while other known repeats of the *B. vulgaris *genome are underrepresented. According to the copy numbers within the *c*_0_*t-1 *library, the satellite pBV is the most abundant satellite family in the genome of *B. vulgaris *followed by the pEV satellite family. This observation is consistent with the prediction that the number of copies of a repeat family in *c*_0_*t *DNA correlates with its abundance in the genome [8].

So far, *c*_0_*t *DNA isolation has been performed in several plant genomes. *c*_0_*t *DNA libraries representing highly repetitive sequences were generated from genomic DNA of *S. bicolor*, *M. acuminata *and *L. triticoides *[8,11,12] while moderately repetitive DNA fractions were isolated from *S. bicolor *and *Z. mays *[8,10]. The *c*_0_*t *analysis enabled the identification of novel repeats, as well as the detection of most abundant repeat classes within a plant genome. *c*_0_*t-1 *DNA analysis performed in the *L. triticoides *genome revealed a highly abundant satellite family [12] which is similar to the observation that most *c*_0_*t-1 *clones of *B. vulgaris *belong to satellite DNA. In contrast, the most abundant repeats detected in the *c*_0_*t *libraries of *S. bicolor*, *M. acuminata *and *Z. mays *belong to retrotransposons or retrotransposon-derived sequences. No significant number of tandemly repeated sequences (except ribosomal genes in the *M. acuminata *and *S. bicolor *genome) has been observed indicating that retrotransposons constitute the main repetitive fraction in these genomes [8,10,11].

The detection of the relatively low number of Miniature inverted-repeat transposable elements (MITEs) in the *c*_0_*t *library of *B. vulgaris *is in contrast to the large number of MITEs that has been described [37] and indicates a possible bias during library construction. A possible reason for the low frequency of MITEs in *c*_0_*t-1 *DNA might be related to the intramolecule renaturation via terminal inverted repeats (TIRs) of single stranded sequences containing MITEs. TIRs of MITEs in *B. vulgaris *are relatively short [37] and *c*_0_*t *clones containing inserts less than 50 bp have been excluded, hence, short MITE sequences have been escaped from analysis.

A possible explanation for the differences in the number of organelle-derived sequences within *c*_0_*t *libraries might be related to plastid and mitochondrial DNA which was isolated together with nuclear DNA. Hribová et al. (2007) and Yuan et al. (2003) isolated the *c*_0_*t-0.05 *DNA and the *c*_0_*t*-*100 *fraction from the *M. acuminata *and *Z. mays *genome, respectively, using a similar approach as in this study [10,11]. The proportion of chloroplast DNA in the *c*_0_*t-0.05 *DNA fraction of *M. acuminata *is 4.2%, which is approximately a third compared to the *c*_0_*t-1 *DNA fraction of *B. vulgaris *and the proportion of organelle-derived DNA in the *c*_0_*t*-100 fraction of *Z. mays *is 1.7% which is much lower as in *c*_0_*t-1 *DNA fraction of *B. vulgaris*. No chloroplast DNA was detectable in the highly repetitive *c*_0_*t *fraction of *S. bicolor *while 10% chloroplast-derived sequences have been observed in the moderate *c*_0_*t *fraction of *S. bicolor *[8,10,11]. Another possible scenario explaining these differences is that chloroplast DNA was integrated into nuclear DNA and consequently *c*_0_*t *sequences with homology to chloroplast DNA might also originate from the nucleus. Chloroplast DNA can be found interspersed into nuclear DNA in many plant species including *B. vulgaris *[42-44]. Moreover, it has been assumed that chloroplast DNA incorporation into the nucleus is a frequent evolutionary event [44]. However, it is very likely that the *B. vulgaris c*_0_*t-1 *clones containing chloroplast sequences originate from contamination of the genomic DNA used for reassociation.

Macas et al. (2007) performed an analysis of genomic sequence data originating from a single 454-sequencing run of the *Pisum sativum *genome to reconstruct the major repeat fraction and identified retroelements as the most abundant repeat class within the genome [19]. Similar analyses investigating crop genome compositions based on next generation sequence technologies have been reported [45,46]. In our study *c*_0_*t-1 *DNA isolation was used for the classification of the major repeat families within the *B. vulgaris *genome and satellite DNA was identified as a highly abundant repeat class. In contrast to genome sequencing projects reflecting the whole genome in its native composition, *c*_0_*t-1 *DNA isolation represents only the repetitive fraction and enables therefore the targeted isolation of major repeats. Furthermore, less sequence data is necessary for the detection of major repeats using *c*_0_*t *DNA isolation compared with next generation sequence reads. We used only 442 kB (0.06% of the genome) sequence data for the detection of the major repeat families of the *B. vulgaris *genome while 33.3 Mb (0.77%) of *P. sativum *[19], 58.91 Mb (1%) of barley [46] and 78.54 Mb (7%) of soybean [45] were analyzed to detect the repeat composition. Therefore, *c*_0_*t *DNA isolation is a very efficient method for the identification of the repetitive DNA of genomes not sequenced yet.

Macas et al. (2007) identified 17 novel tandem repeat families, and two minisatellites were physically mapped on *P. sativum *chromosomes [19]. In order to demonstrate the potential of the *c*_0_*t-1 *DNA library for the detection of novel repeat classes we focused on the identification of tandemly repeated sequences, particularly on the identification of minisatellites. So far, the targeted isolation of minisatellites from plant genomes has not been described and this repeat type is only poorly characterized. It is not feasible to isolate most minisatellites as restriction satellites because of their short length, unusual base composition and hence, absence of recognition sites. The identification of nine minisatellite families as described here shows the potential of *c*_0_*t *DNA analysis for the rapid and targeted isolation of minisatellites from genomes. In addition we identified three satellite families undiscovered yet because of their moderate abundance.

In contrast to typical G/C-rich minisatellites [13], all nine *B. vulgaris *families show a low G/C content: six of the nine families have a G/C-content between 24% to 33% (Table 1). Repetitive sequences are often subject to modification by cytosine methylation. It is known that deamination converts 5-methylcytosine to thymine, resulting in an increased AT-content [47]. This might be a possible reason of the low G/C level of *B. vulgaris *minisatellites. Furthermore, the monomers of the *B. vulgaris *minisatellite families are different in sequence length and nucleotide composition from the 14 to 16 bp G/C-rich core sequence of minisatellites in *A. thaliana *or human [25,26].

Most conventional plant satellites show a low G/C content [48]. However, the *Fok*I-satellite has a G/C content of 60% which is in contrast to the *Hin*fI-satellite and *Alu*I-satellite and other satellites described in *B. vulgaris*. Moreover, the monomer size of 130 bp of the *Fok*I-satellite is different from the typical monomer size of plant satellites of 160-180 bp or 320 to 370 bp [15], whereas monomers of *Hinf*I-satellite and *Alu*I-satellite fall into the typical monomer size range.

Only two of the nine minisatellite families (BvMSat03 and BvMSat04) show the typical ladder-like pattern in Southern analyses. Dimers of BvMSat03 were detectable after restriction of genomic DNA with *Bsm*AI (Figure 2B, lane 2). However, partial restriction with *Bsm*AI generates di- to decamers of BvMSat03 (not shown), indicating the highly conserved recognition site of *Bsm*AI in BvMSat03-monomers.

Hybridization of minisatellites to *Msp*I and *Hpa*II digested DNA indicates cytosine methylation of the recognition site CCGG. The *Hin*fI-satellite and *Alu*I-satellite family show also a strong methylation, while a reduced CNG methylation was detectable for some *Fok*I-satellite copies. This might be an indication that some *Fok*I-satellite copies lacking CNG methylation might be linked to the activation of transcription or to chromatin remodeling [49-52].

Little is known about the localization of minisatellites on plant chromosomes. So far, only two minisatellite families were physically mapped on chromosomes of *P. sativum *using FISH [19]. In contrast to minisatellites of *P. sativum *detectable only on one and two chromosome pairs [19], respectively, the *B. vulgaris *minisatellites were detectable mostly on all 18 chromosomes with different signal strength, preferentially distributed in the intercalary heterochromatin and terminal chromosome regions. This pattern of chromosomal localization shows similarity to the distribution of microsatellite sequences on *B. vulgaris *chromosomes, which show a dispersed organization along chromosomes including telomeres and intercalary chromosomal regions, but are mostly excluded from the centromere [36]. This is in contrast to the chromosomal localization of the highly abundant satellite families pBV and pEV and the satellite family pAv34 [33], which are detectable in large tandem arrays in centromeric/pericentromeric, intercalary and subtelomeric regions, respectively. Only BvMSat08 and BvMSat09 can be found in large tandem array blocks within the intercalary heterochromatin.

The *Fok*I, *Alu*I and *Hin*fI satellite families show dispersed localization in smaller arrays with different array sizes among chromosomes, preferentially in the intercalary heterochromatin and in terminal chromosome regions, respectively. The *Hin*fI-satellite is predominantly distributed in terminal chromosome regions. The pAv34 satellite is also localized in subtelomeric chromosome positions [33]. However, no copies of pAv34 were detected within the 13 kb BAC [EMBL:DQ374018] and the 11 kb BAC [EMBL:DQ374019] that contain a tandem array of the *Hin*fI-satellite consisting of 14 and 26 monomers, respectively, indicating no interspersion of both satellite families. High resolution FISH on pachytene chromosomes or chromatin fibers using probes of pAv34 and the *Hin*fI-satellite could be used to gain information about possible interspersion or physically neighborhood of both satellite families.

Because of their small size (2-3 μm) and similar morphology (most chromosomes are meta- to submetacentric) FISH karyotype analysis of *B. vulgaris *has not been established yet. In contrast to conventional staining techniques [53], which are not efficient for reliable karyotyping of small chromosomes, FISH is an applicable method for the discrimination of the *B. vulgaris *chromosomes. Chromosome 1 can be identified by strong signals of terminal 18S-5.8S-25S rRNA genes while chromosome 4 is detectable by 5S rRNA hybridization patterns [54]. FISH using probes of BvMSat08 enables the identification of another chromosome pair, due to the localization of the large BvMSat08 blocks on both chromosome arms. Hence, this minisatellite may be an important cytogenetic marker for future karyotyping based on FISH. Also, because of their specific chromosomal localization, the minisatellite BvMSat09, the *Alu*I satellite and the *Hin*fI satellite can serve as cytogenetic markers and support FISH karyotyping in *B. vulgaris*.

It has been reported that human minisatellites originated from retroviral LTR-like sequences or from the 5' end of Alu elements [55,56] but also other scenarios of the origin and the evolution were described in human and in primates [57,58]. In plants, only few data are available about the origin and the evolution of minisatellite sequences. We propose a possible process which might describe the origin and/or evolution of minisatellites from microsatellites in the genome of *B. vulgaris*. Sequence analysis suggests that BvMSat07 originated from a microsatellite with the 5 bp monomer sequence GATCA. During microsatellite evolution complex arrays of six monomers evolved, which were subsequently tandemly arranged. The resulting minisatellite is 30 bp in size and consists of one GAAAA, AATAA and GTTCA and three adjacent GATCA monomers. The 5 bp subrepeats differing from the GATCA monomer sequence might have originated from the GATCA-motif by point mutation. The complex repeat shows structural similarities to higher-order structures of satellites, e.g. the human alpha satellite [59]. A satellite higher-order structure is defined as monomers which form tandemly arranged highly homogenous multimeric repeat units [59]. One complex repeat of the microsatellite might have been duplicated and enlarged by replication slippage resulting in a BvMSat07 array (Figure 4) and its copy number might have been increased by recombination between homologous loci.

Another scenario of minisatellite origin and array enlargement can be concluded from the minisatellite family BvMSat05. The palindromic sequences within the highly conserved 50 bp sequence adjacent to BvMSat05 arrays may form secondary DNA structures, which may interfere with the DNA polymerase during DNA replication. This may result in slippage replication of the DNA motif upstream, contributing to the generation and enlargement of BvMSat05 arrays. Moreover, FISH revealed a subtelomeric localization of BvMSat05 clusters on some chromosomes, hence, the head to head junction of head to tail arrays typical for BvMSat05 may result from breakage-fusion-bridge cycles as postulated for tandem repeats near at terminal regions of rye chromosomes [60]. It has been reported that palindromic sequences may induce genomic instability through provoking double strand breaks and recombination [61]. Therefore, the head to head junction may also be the result of DNA repair following possible double strand breaks within BvMSat05 arrays.

It has also been discussed that tandemly repeated sequences are derived from 3' UTR regions of retrotransposons [62]. Analysis of retrotransposons in *B. vulgaris *[40,63,64] did not reveal any homology to minisatellite arrays or adjacent regions. However, we detected LTR sequences of a yet uncharacterized retrotransposon in the close vicinity of BvMSat04 arrays (not shown). Therefore, the evolution and dispersion of BvMSat04 arrays within the *B. vulgaris *genome might also be the result of the activity of this retrotransposon.

In this study we focused in detail on the characterization of novel minisatellites and satellites. Nevertheless, these tandem repeats make up only 6.8% of the 517 uncharacterized *c*_0_*t-1 *sequences indicating that the *c*_0_*t-1 *library is an efficient source for the identification of further repeat classes. Examples are the 118 *c*_0_*t-1 *sequences possessing motifs of retrotransposon families as well as the identification of the *envelope*-like *Copia *element Cotzilla [40].

## Conclusions

We isolated highly to moderately repetitive DNA sequences from *B. vulgaris *originating from a *c*_0_*t-1 *DNA library. Providing the first comprehensive classification of repeats, we observed that the satellites pBV and pEV form the most abundant repeat families in *B. vulgaris*.

We identified nine minisatellite and three previously unknown satellite families demonstrating that the analysis of *c*_0_*t-1 *DNA is an efficient method for the rapid and targeted isolation of tandemly repeated sequences, particularly of minisatellites from plant genomes. Minisatellites in *B. vulgaris *display a low G/C content and deviate strongly from the G/C-rich minisatellite core sequence observed in *A. thaliana *and human [25,26] showing that a minisatellite core motif is not conserved in plant genomes. Physical mapping of the minisatellites on chromosomes using FISH revealed a mainly dispersed chromosomal distribution pattern. The possible origin, enlargement and amplification of minisatellites arrays were concluded for some minisatellite families. Complex structures of microsatellite arrays may play a role for the generation of minisatellites. Moreover, DNA sequences that contain palindromic motifs may be linked to slippage replication due to interfering with DNA polymerase during replication and may therefore be involved in the origin of minisatellites.

## Methods

### Plant material and DNA preparation

Plants of *Beta vulgaris *ssp. *vulgaris *genotype KWS2320 were grown under greenhouse conditions. Genomic DNA was isolated from young leaves using the CTAB (cetyltrimethyl/ammonium bromide) standard protocol [65].

### Construction of the *c*_0_*t-1 *DNA library

The *c*_0_*t-1 *DNA was prepared with some modifications according to Zwick et al. [9]. 640 μg of genomic DNA was dissolved in 1600 μl water and sheared at 99°C for 10 minutes followed by sonication at 80°C for 3 minutes to generate DNA fragments ranging in size predominantly between 0.5 to 1.0 kb. Renaturation of DNA fragments was carried out in a 0.3 M NaCl solution at 65°C after initial denaturation at 92°C for 10 minutes. The renaturation time was calculated according to Zwick et al. [9]. S1 nuclease treatment followed to remove single stranded DNA and single strand overhangs on renaturated double stranded DNA. The enzyme was inactivated by adding stop solution (3 M Tris pH 8.0, 0.5 M EDTA) according to Ostermeier et al. [66] and incubation at 72°C for 20 min. Blunt end *c*_0_*t-1 *DNA fragments were ligated into the *Sma*I site of dephosphorylated pUC18 vector. After transformation of XL1Blue cells (Stratagene), positive clones were identified by blue/white screening and transferred into 384-well plates, grown in LB freezing medium and stored at -80°C.

### Sequencing of *c*_0_*t-1 *clones

Clones were grown in Terrific Broth (TB) medium (1.2% peptone, 2.4% yeast extract, 72 mM K_2_HPO_4_, 17 mM KH_2_PO_4 _and 0.4% glycerol) including 100 μg/ml ampicillin at 37°C. Small-scale plasmid isolation was performed by the TELT procedure [67]. Plasmids were sequenced on an ABI 3730XL sequencer (Applied Biosystems; Foster City, CA/USA) using BigDye terminator chemistry, in forward (5'-CGTTGTAAAACGACGGCCAGT-3') and/or reverse (5'-CAGGAAACAGCTATGACCATG-3') directions.

### Computational methods

Sequences in *c*_0_*t-1 *DNA library, which are homologous to previously characterized *B. vulgaris *repeats, were identified using local BLAST option of the *BioEdit *software [68] with a representative query sequence of the repeat family. Novel *c*_0_*t-1 *DNA sequences were characterized using the EMBL database homology search against nucleotide and amino acid sequences and an e-value threshold of 10^-3^. The remaining fraction of the *c*_0_*t-1 *DNA without homology to EMBL database entries was used for the identification of tandem repeats using *Tandem Repeats Finder *[69]. Subsequently, c_0_*t-1 *sequences containing tandem repeats were used as query sequence for the identification of further DNA copies from BAC end sequences [39], (Holtgräwe and Weisshaar, in preparation) to reveal their abundance and array structures. The DNA sequences of each tandem repeat family were aligned manually using the *Phylogenetic Data Editor *[70]. The detection of G/C content and identity values of each tandem repeat family was determined by a *G/C Content Calculator *and *ClustalX *[71] using at least 20 randomly selected monomers of representative tandem arrays. Sequences contigs have been established using *DNASTAR Lasergene v8.0*.

### PCR conditions

Primer pairs were derived from conserved regions of minisatellite and satellite monomers. The PCR reactions with 50 ng genomic DNA and a final primer concentration of 0.5 μM were performed in a 20 μl volume containing 0.2 mM dNTPs and 1 unit of GoTaq polymerase (Promega). The PCR conditions were 94°C for 3 min, followed by 30 cycles of 94°C for 30 s, 47°C to 65°C depending on the primer melting temperature of each repeat family, for 30 s, 72°C for 40 s and a final incubation at 72°C for 5 min. For the generation of probes for Southern hybridization and fluorescent *in situ *hybridization, the same primers or M13 primers were used to amplify tandem repeats from *c*_0_*t-1 *clones.

### Southern hybridization

For Southern hybridization 5 μg of genomic DNA was restricted with different enzymes, separated on 1.2% agarose gels and transferred onto Hybond-XL nylon membranes (GE Healthcare) using alkaline transfer. Southern hybridizations using ^32^P-labelled probes were performed using standard protocols [72]. Filters were hybridized at 60°C and washed at 60°C in 2× SSC/0.1% SDS for 3 h. The signals were detected by autoradiography.

### FISH

The meristem of young leaves was used for the preparation of mitotic chromosomes. The maceration of plant material was performed in an enzyme mixture consisting of 0.3% (w/v) cytohelicase (Sigma), 1.8% (w/v) cellulase from *Aspergillus niger *(Sigma), 0.2% (w/v) cellulase Onozuka-R10 (Serva) and 20% (v/v) pectinase from *A. niger*; followed by spreading of nuclei on slides. Probes of tandem repeats were labelled with biotin-16-dUTP (Roche) by PCR according to Schwarzacher et al. [73] while 18S-5.8S-25S rRNA genes were labelled by nick-translation with digoxygenin-11-dUTP (Roche). The hybridization and detection were performed according to Schmidt et al. [54]. Chromosome preparations were counterstained with DAPI (4',6'-diamino-2-phenylindole) and mounted in antifade solution (CitiFluor). The examination of slides was carried out with a Zeiss Axioplan2 Imaging fluorescent microscope with filters 09 (FITC), 15 (Cy3) and 02 (DAPI). The images were acquired with the Applied Spectral Imaging v. 3.3 software coupled with the high-resolution CCD camera ASI BV300-20A. The contrast of images was optimized using only functions affecting whole image equally by Adobe Photoshop 7.0 software.

## Authors' contributions

FZ and TW wrote the paper, participated in the bioinformatic analyses of the *c*_0_*t-1 *library, carried out the alignments of the tandemly repeated *c*_0_*t-1 *sequences and performed the molecular genetic studies. DH and BW performed the sequencing of the *c*_0_*t-1 *clones, provided the BAC end sequence database and helped to draft the manuscript. TS participated in the design and coordination of the project and has been involved in the writing of the article. All authors read and approved the final manuscript.

## References

[B1] Britten RJ Kohne DE Repeated sequences in DNA. Hundreds of thousands of copies of DNA sequences have been incorporated into the genomes of higher organisms Science 1968 161 841 529 540 10.1126/science.161.3841.529 4874239

[B2] Ugarkovic D Functional elements residing within satellite DNAs Embo Reports 2005 6 11 1035 1039 10.1038/sj.embor.7400558 16264428 PMC1371040

[B3] Lisch D Epigenetic regulation of transposable elements in plants Annu Rev Plant Biol 2009 60 43 66 10.1146/annurev.arplant.59.032607.092744 19007329

[B4] Feschotte C Transposable elements and the evolution of regulatory networks Nat Rev Genet 2008 9 5 397 405 10.1038/nrg2337 18368054 PMC2596197

[B5] Lippman Z Gendrel AV Black M Vaughn MW Dedhia N McCombie WR Lavine K Mittal V May B Kasschau KD Role of transposable elements in heterochromatin and epigenetic control Nature 2004 430 6998 471 476 10.1038/nature02651 15269773

[B6] Weil C Martienssen R Epigenetic interactions between transposons and genes: lessons from plants Curr Opin Genet Dev 2008 18 2 188 192 10.1016/j.gde.2008.01.015 18339541

[B7] Jurka J Kapitonov VV Kohany O Jurka MV Repetitive Sequences in Complex Genomes: Structure and Evolution Annu Rev Genomics Hum Genet 2006 8 241 259 10.1146/annurev.genom.8.080706.092416 17506661

[B8] Peterson DG Schulze SR Sciara EB Lee SA Bowers JE Nagel A Jiang N Tibbitts DC Wessler SR Paterson AH Integration of Cot analysis, DNA cloning, and high-throughput sequencing facilitates genome characterization and gene discovery Genome Res 2002 12 5 795 807 10.1101/gr.226102 11997346 PMC186575

[B9] Zwick MS Hanson RE McKnight TD IslamFaridi MN Stelly DM Wing RA Price HJ A rapid procedure for the isolation of C(0)t-1 DNA from plants Genome 1997 40 1 138 142 10.1139/g97-020 18464813

[B10] Yuan Y SanMiguel PJ Bennetzen JL High-Cot sequence analysis of the maize genome Plant J 2003 34 2 249 255 10.1046/j.1365-313X.2003.01716.x 12694599

[B11] Hribová E Dolezelová M Town CD Macas J Dolezel J Isolation and characterization of the highly repeated fraction of the banana genome Cytogenet Genome Res 2007 119 3-4 268 274 10.1159/000112073 18253041

[B12] Anamthawat-Jónsson K Wenke T Thórsson Æ Th Sveinsson S Zakrzewski F Schmidt T Evolutionary diversification of satellite DNA sequences from Leymus (Poaceae: Triticeae) Genome 2009 52 381 390 10.1139/G09-013 19370093

[B13] Vergnaud G Denoeud F Minisatellites: mutability and genome architecture Genome Res 2000 10 7 899 907 10.1101/gr.10.7.899 10899139

[B14] Chistiakov DA Hellemans B Volckaert FAM Microsatellites and their genomic distribution, evolution, function and applications: A review with special reference to fish genetics Aquaculture 2006 255 1-4 1 29 10.1016/j.aquaculture.2005.11.031

[B15] Hemleben V Kovarik A Torres-Ruiz RA Volkov RA Beridze T Plant highly repeated satellite DNA: molecular evolution, distribution and use for identification of hybrids Systematics and Biodiversity 2007 5 03 277 289 10.1017/S147720000700240X

[B16] Haber JE Louis EJ Minisatellite origins in yeast and humans Genomics 1998 48 1 132 135 10.1006/geno.1997.5153 9503027

[B17] Taylor JS Breden F Slipped-strand mispairing at noncontiguous repeats in Poecilia reticulata: a model for minisatellite birth Genetics 2000 155 3 1313 1320 10880490 10.1093/genetics/155.3.1313 PMC1461170

[B18] Schmidt A Doudrick RL Heslop-Harrison JS Schmidt T The Contribution of Short Repeats of Low Sequence Complexity to Large Conifer Genomes Theor Appl Genet 2000 101 7 14 10.1007/s001220051442

[B19] Macas J Neumann P Navratilova A Repetitive DNA in the pea (Pisum sativum L.) genome: comprehensive characterization using 454 sequencing and comparison to soybean and Medicago truncatula BMC Genomics 2007 8 1 427 443 10.1186/1471-2164-8-427 18031571 PMC2206039

[B20] Gustafson JP Yano M Genetic mapping of hypervariable minisatellite sequences in rice (Oryza sativa L.) Theor Appl Genet 2000 100 3 447 453 10.1007/s001220050058

[B21] Broun P Tanksley SD Characterization of tomato DNA clones with sequence similarity to human minisatellites 33.6 and 33.15 Plant Mol Biol 1993 23 2 231 242 10.1007/BF00029000 8219062

[B22] Hisatomi Y Hanada K Iida S The retrotransposon RTip1 is integrated into a novel type of minisatellite, MiniSip1, in the genome of the common morning glory and carries another new type of minisatellite, MiniSip2 Theor Appl Genet 1997 95 7 1049 1056 10.1007/s001220050661

[B23] Martienssen RA Baulcombe DC An unusual wheat insertion sequence (WIS1) lies upstream of an a-amylase gene in hexaploid wheat, and carries a "minisatellite" array Mol Gen Genet 1989 217 2 401 410 10.1007/BF02464910 2549380

[B24] Somers DJ Zhou Z Bebeli PJ Gustafson JP Repetitive, genome-specific probes in wheat (Triticum aestivum L. em Thell) amplified with minisatellite core sequences Theor Appl Genet 1996 93 5 982 989 10.1007/BF00224102 24162434

[B25] Tourmente S Identification of new minisatellites loci in Arabidopsis thaliana J Exp Bot 1998 49 318 21 25 10.1093/jexbot/49.318.21

[B26] Tourmente S Deragon JM Lafleuriel J Tutois S Pélissier T Cuvillier C Espagnol MC Picard G Characterization of minisatellites in Arabidopsis thaliana with sequence similarity to the human minisatellite core sequence Nucleic Acids Res 1994 22 16 3317 3321 10.1093/nar/22.16.3317 8078766 PMC523724

[B27] Arumuganathan K Earle ED Nuclear DNA content of some important plant species Plant Mol Bio Reporter 1991 9 3 186 198 10.1007/BF02672064

[B28] Flavell RB Bennett MD Smith JB Smith DB Genome Size and Proportion of Repeated Nucleotide-Sequence DNA in Plants Biochemical Genetics 1974 12 4 257 269 10.1007/BF00485947 4441361

[B29] PROJECT: Generation of a physical, BAC-based map of the sugar beet (Beta vulgaris) genome (GABI-BPM) http://www.genomforschung.uni-bielefeld.de/GF-research/GABI-BPM.html

[B30] Schmidt T Metzlaff M Cloning and Characterization of a Beta-vulgaris Satellite DNA Family Gene 1991 101 2 247 250 10.1016/0378-1119(91)90418-B 2055488

[B31] Menzel G Dechyeva D Wenke T Holtgräwe D Weisshaar B Schmidt T Diversity of a Complex Centromeric Satellite and Molecular Characterization of Dispersed Sequence Families in Sugar Beet (Beta vulgaris) Ann Bot (Lond) 2008 102 521 530 10.1093/aob/mcn131 PMC2701778 18682437

[B32] Schmidt T Jung C Metzlaff M Distribution and evolution of two satellite DNAs in the genus Beta Theor Appl Genet 1991 82 793 799 10.1007/BF00227327 24213457

[B33] Dechyeva D Schmidt T Molecular organization of terminal repetitive DNA in Beta species Chromosome Res 2006 14 8 881 897 10.1007/s10577-006-1096-8 17195925

[B34] Schmidt T Heslop-Harrison JS Variability and evolution of highly repeated DNA sequences in the genus Beta Genome 1993 36 6 1074 1079 10.1139/g93-142 8112571

[B35] Schmidt T Heslop-Harrison JS Genomes, genes and junk: the large-scale organization of plant chromosomes Trends in Plant Science 1998 3 5 195 199 10.1016/S1360-1385(98)01223-0

[B36] Schmidt T Heslop-Harrison JS The physical and genomic organization of microsatellites in sugar beet Proc Natl Acad Sci USA 1996 93 16 8761 8765 10.1073/pnas.93.16.8761 8710945 PMC38747

[B37] Menzel G Dechyeva D Keller H Lange C Himmelbauer H Schmidt T Mobilization and evolutionary history of miniature inverted-repeat transposable elements (MITEs) in Beta vulgaris L Chromosome Res 2006 14 8 831 844 10.1007/s10577-006-1090-1 17171577

[B38] Jacobs G Dechyeva D Menzel G Dombrowski C Schmidt T Molecular characterization of Vulmar1, a complete mariner transposon of sugar beet and diversity of mariner- and En/Spm-like sequences in the genus Beta Genome 2004 47 6 1192 1201 10.1139/g04-067 15644978

[B39] McGrath JM Shaw RS de los Reyes BG Weiland JJ Construction of a sugar beet BAC library from a hybrid with diverse traits Plant Mol Bio Reporter 2004 22 1 23 28 10.1007/BF02773345

[B40] Weber B Wenke T Frommel U Schmidt T Heitkam T The Ty1-copia families SALIRE and Cotzilla populating the Beta vulgaris genome show remarkable differences in abundance, chromosomal distribution, and age Chromosome Res DOI: 10.1007/s10577-009-9104-4 10.1007/s10577-009-9104-4 20039119

[B41] Zilberman D Henikoff S Genome-wide analysis of DNA methylation patterns Development 2007 134 22 3959 3965 10.1242/dev.001131 17928417

[B42] Cullis CA Vorster BJ Vyver C Van Der Kunert KJ Transfer of genetic material between the chloroplast and nucleus: how is it related to stress in plants? Ann Bot (Lond) 2009 103 4 625 633 10.1093/aob/mcn173 PMC2707348 18801916

[B43] Blanchard JL Schmidt GW Pervasive migration of organellar DNA to the nucleus in plants J Mol Evol 1995 41 4 397 406 10.1007/BF00160310 7563126

[B44] Ayliffe MA Timmis JN Scott NS Homologies to chloroplast DNA in the nuclear DNA of a number of Chenopod species Theor Appl Genet 1988 75 2 282 285 10.1007/BF00303965

[B45] Swaminathan K Varala K Hudson ME Global repeat discovery and estimation of genomic copy number in a large, complex genome using a high-throughput 454 sequence survey BMC Genomics 2007 8 132 10.1186/1471-2164-8-132 17524145 PMC1894642

[B46] Wicker T Taudien S Houben A Keller B Graner A Platzer M Stein N A whole-genome snapshot of 454 sequences exposes the composition of the barley genome and provides evidence for parallel evolution of genome size in wheat and barley Plant J 2009 59 5 712 722 10.1111/j.1365-313X.2009.03911.x 19453446

[B47] Montero LM Filipski J Gil P Capel J Martinez-Zapater JM Salinas J The distribution of 5-methylcytosine in the nuclear genome of plants Nucleic Acids Res 1992 20 12 3207 3210 10.1093/nar/20.12.3207 1620618 PMC312460

[B48] Macas J Mészáros T Nouzová M PlantSat: a specialized database for plant satellite repeats Bioinformatics 2002 18 1 28 35 10.1093/bioinformatics/18.1.28 11836208

[B49] Bartee L Malagnac F Bender J Arabidopsis cmt3 chromomethylase mutations block non-CG methylation and silencing of an endogenous gene Genes Dev 2001 15 14 1753 1758 10.1101/gad.905701 11459824 PMC312734

[B50] Jackson JP Lindroth AM Cao X Jacobsen SE Control of CpNpG DNA methylation by the KRYPTONITE histone H3 methyltransferase Nature 2002 416 6880 556 560 10.1038/nature731 11898023

[B51] Johnson LM Cao XF Jacobsen SE Interplay between two epigenetic marks: DNA methylation and histone H3 lysine 9 methylation Current Biology 2002 12 16 1360 1367 10.1016/S0960-9822(02)00976-4 12194816

[B52] Lindroth AM Cao X Jackson JP Zilberman D McCallum CM Henikoff S Jacobsen SE Requirement of CHROMOMETHYLASE3 for maintenance of CpXpG methylation Science 2001 292 5524 2077 2080 10.1126/science.1059745 11349138

[B53] Jong JH Bock TSM Use of haploids of Beta vulgaris L. for the study of orcein and giemsa stained chromosomes Euphytica 1978 27 1 41 47 10.1007/BF00039118

[B54] Schmidt T Schwarzacher T Heslop-Harrison JS Physical mapping of rRNA genes by fluorescent in-situ hybridization and structural analysis of 5S rRNA genes and intergenic spacer sequences in sugar beet (Beta vulgaris) Theor Appl Genet 1994 88 6 629 636 10.1007/BF01253964 24186156

[B55] Armour JA Wong Z Wilson V Royle NJ Jeffreys AJ Sequences flanking the repeat arrays of human minisatellites: association with tandem and dispersed repeat elements Nucleic Acids Res 1989 17 13 4925 4935 10.1093/nar/17.13.4925 2762114 PMC318084

[B56] Jurka J Gentles AJ Origin and diversification of minisatellites derived from human Alu sequences Gene 2006 365 21 26 10.1016/j.gene.2005.09.029 16343813

[B57] Barros P Blanco MG Boan F Gomez-Marquez J Evolution of a complex minisatellite DNA sequence Mol Phylogenet Evol 2008 49 2 488 494 10.1016/j.ympev.2008.07.021 18723095

[B58] Boan F Blanco MG Quinteiro J Mourino S Gomez-Marquez J Birth and evolutionary history of a human minisatellite Mol Biol Evol 2004 21 2 228 235 10.1093/molbev/msh007 14595097

[B59] Rudd MK Willard HF Analysis of the centromeric regions of the human genome assembly Trends Genet 2004 20 11 529 533 10.1016/j.tig.2004.08.008 15475110

[B60] Vershinin AV Schwarzacher T Heslop Harrison JS The Large-Scale Genomic Organization of Repetitive DNA Families at the Telomeres of Rye Chromosomes Plant Cell 1995 7 11 1823 1833 10.1105/tpc.7.11.1823 8535136 PMC161041

[B61] Lisnic B Svetec IK Stafa A Zgaga Z Size-dependent palindrome-induced intrachromosomal recombination in yeast DNA Repair (Amst) 2009 8 3 383 389 10.1016/j.dnarep.2008.11.017 19124276

[B62] Macas J Koblizkova A Navratilova A Neumann P Hypervariable 3' UTR region of plant LTR-retrotransposons as a source of novel satellite repeats Gene 2009 448 2 198 206 10.1016/j.gene.2009.06.014 19563868

[B63] Wenke T Holtgrawe D Horn AV Weisshaar B Schmidt T An abundant and heavily truncated non-LTR retrotransposon (LINE) family in Beta vulgaris Plant Mol Biol 2009 71 585 597 10.1007/s11103-009-9542-6 19697140

[B64] Heitkam T Schmidt T BNR - a LINE family from Beta vulgaris - contains a RRM domain in open reading frame 1 and defines a L1 sub-clade present in diverse plant genomes Plant J 2009 59 6 872 882 10.1111/j.1365-313X.2009.03923.x 19473321

[B65] Saghai-Maroof MA Soliman KM Jorgensen RA Allard RW Ribosomal DNA spacer-length polymorphisms in barley: mendelian inheritance, chromosomal location, and population dynamics Proc Natl Acad Sci USA 1984 81 24 8014 8018 10.1073/pnas.81.24.8014 6096873 PMC392284

[B66] Ostermeier M Nixon AE Shim JH Benkovic SJ Combinatorial protein engineering by incremental truncation Proc Natl Acad Sci USA 1999 96 7 3562 3567 10.1073/pnas.96.7.3562 10097076 PMC22333

[B67] Ausubel FM Brent R Kingston RE Moore DD Seidman JG Smith JAKS Current protocols in molecular biology 1987 New York: N.Y: John Wiley & Sons, Inc

[B68] Hall TA BioEdit: a user-friendly biological sequence alignment editor and analysis program for Windows 95/98/NT Nucleic Acids Symposium Series 1999 41 95 98

[B69] Benson G Tandem repeats finder: a program to analyze DNA sequences Nucleic Acids Res 1999 27 2 573 580 10.1093/nar/27.2.573 9862982 PMC148217

[B70] The Phylogenetic Data Editor http://www.phyde.de

[B71] Larkin MA Blackshields G Brown NP Chenna R McGettigan PA McWilliam H Valentin F Wallace IM Wilm A Lopez R Clustal W and Clustal X version 2.0 Bioinformatics 2007 23 21 2947 2948 10.1093/bioinformatics/btm404 17846036

[B72] Sambrook J Fritsch EF Maniatis T Molecular Cloning: A Laboratory Manual 1989 2 Cold Spring Harbor Laboratory Press, Cold Spring Harbor, NY

[B73] Schwarzacher T Heslop-Harrison P Practical in situ hybridization 2000 Oxford: Bios Scientific Publishers

